# The decatenation checkpoint

**DOI:** 10.1038/sj.bjc.6603537

**Published:** 2007-01-09

**Authors:** M Damelin, T H Bestor

**Affiliations:** 1 Department of Genetics and Development, College of Physicians and Surgeons of Columbia University, 701 W. 168th St., New York, NY 10032, USA

**Keywords:** decatenation checkpoint, G_2_ checkpoint, cancer stem cell, topoisomerase II, ICRF-193

## Abstract

The decatenation checkpoint delays entry into mitosis until the chromosomes have been disentangled. Deficiency in or bypass of the decatenation checkpoint can cause chromosome breakage and nondisjunction during mitosis, which results in aneuploidy and chromosome rearrangements in the daughter cells. A deficiency in the decatenation checkpoint has been reported in lung and bladder cancer cell lines and may contribute to the accumulation of chromosome aberrations that commonly occur during tumour progression. A checkpoint deficiency has also been documented in cultured stem and progenitor cells, and cancer stem cells are likely to be derived from stem and progenitor cells that lack an effective decatenation checkpoint. An inefficient decatenation checkpoint is likely to be a source of the chromosome aberrations that are common features of most tumours, but an inefficient decatenation checkpoint in cancer stem cells could also provide a potential target for chemotherapy.

## FUNCTION OF THE DECATENATION CHECKPOINT

**Figure 1:**
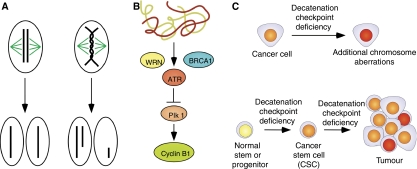
(**A**) Mitosis in the presence of entangled chromosomes leads to aneuploidy. The decatenation checkpoint guards against nondisjunction and chromosome breakage that occur when the cell enters mitosis before the chromosomes have been sufficiently decatenated by Topo II. (**B**) Working model of decatenation checkpoint signalling. (**C**) Checkpoint deficiency in cancer cells could result in additional chromosome imbalances that increase tumour malignancy (top). The presence of additional chromosome aberrations is represented by darker colour of the nucleus. Decatenation checkpoint deficiency is an inherent feature of stem and progenitor cells and may lead to the formation of cancer stem cells (bottom). Chemotherapy that targets the decatenation checkpoint may efficiently target cancer cells and CSCs.

Entanglements of sister chromatids are a consequence of DNA replication, and nonreplicative catenations arise incidentally during interphase (Gimenez-Abian *et al*, 2000). Gain and loss of whole chromosomes and chromosome fragments result when cells complete mitosis in the presence of entangled chromosomes (Figure 1A). The G_2_ phase decatenation checkpoint delays entry into mitosis until chromosomes have been decatenated (disentangled) by topoisomerase II (topo II) (Downes *et al*, 1994). The decatenation checkpoint is distinct from the DNA damage checkpoint, the chromatin deacetylation checkpoint, the spindle assembly checkpoint and other G_2_/M checkpoints.

Topoisomerase II enzymes disentangle chromosomes by passing one double helix through a transient double-strand break (DSB) in another double helix and then resealing the break. The classical topo II inhibitors, which include etoposide (VP-16), teniposide (VM-26), amsacrine, and adriamycin/doxorubicin, generate massive numbers of DSBs and trigger the G_2_ phase DNA damage checkpoint (Kaufmann and Kies, 1998). Studies with these topo II poisons precluded the discovery of the decatenation checkpoint. In contrast, the catalytic inhibitors, which include ICRF-193 and related bisdioxopiperazines, inhibit topo II before DSB formation and activate the decatenation checkpoint, but not the DNA damage checkpoint (Downes *et al*, 1994; Roca *et al*, 1994). The decatenation checkpoint is typically studied with the bisdioxopiperazine topo II inhibitors.

### CHECKPOINT DEFICIENCY AND CHROMOSOME INSTABILITY

Deficiency in or bypass of the decatenation checkpoint can directly result in chromosome instability because cells can complete division in the presence of entangled chromosomes (Figure 1A). Chromosome breakage and nondisjunction at anaphase leads to chromosome translocations and other aberrations in daughter cells. These chromosome imbalances have been observed in several cell types following bisdioxopiperazine inhibition of topo II (Gorbsky, 1994; Ishida *et al*, 1994; Deming *et al*, 2002; Damelin *et al*, 2005). The spindle checkpoint would be expected to prevent anaphase after failure or bypass of the decatenation checkpoint. However, the spindle checkpoint is inefficient when the decatenation checkpoint has failed. A possible explanation is that the spindle checkpoint is not activated because the tension generated by the chromosome entanglements is not distinguished from the tension generated by amphitelic attachment (Damelin *et al*, 2005). This idea is supported by the result that spindle checkpoint activation in cohesin-deficient cells was relieved by the bisdioxopiperazine inhibition of topo II (Vagnarelli *et al*, 2004). It is also possible that the spindle checkpoint delays cell cycle progression only transiently and is eventually bypassed (Andreassen *et al*, 2003).

Specific types of genetic changes are predicted to result from an inefficient decatenation checkpoint. The gain and loss of whole chromosomes and chromosome fragments are expected, while point mutations and mitotic recombination events are not. An analysis of loss-of-heterozygosity (LOH) mutations found that in mouse embryonic stem (ES) cells, the majority of LOH mutations were attributable to nondisjunction, whereas in mouse embryonic fibroblasts, LOH owing to nondisjunction was not observed (Cervantes *et al*, 2002). The LOH events in ES cells are likely to arise from the decatenation checkpoint deficiency in those cells.

## CHECKPOINT STATUS IN CANCER CELLS

The decatenation checkpoint has been observed in many mammalian cell types. The original report documented a functional checkpoint in the Indian muntjac cells, PtK2 rat kangaroo cells, Chinese hamster ovary cells, SV-MRC5-transformed human fibroblasts, MSU1.1 v-myc-immortalised human fibroblasts, and HeLa human cervical carcinoma cell lines (Downes *et al*, 1994). Subsequently, the checkpoint was observed in human lymphoblastoid cell lines (Deming *et al*, 2001), mouse embryonic fibroblasts (Damelin *et al*, 2005), and primary cultures and strains of human uroepithelial cells (Doherty *et al*, 2003) and human fibroblasts (Kaufmann and Kies, 1998; Deming *et al*, 2001; Franchitto *et al*, 2003; Damelin *et al*, 2005).

It was surprising to find that wild-type stem and progenitor cells have an inherent deficiency in the decatenation checkpoint and complete cell division in the presence of entangled chromosomes (Damelin *et al*, 2005). The deficiency was documented in mouse ES cells, mouse neural progenitors, and human CD34+ haematopoietic progenitors. When ES cells were induced to differentiate, the efficiency of the checkpoint increased, which indicated that the observed deficiency is a feature of the undifferentiated state.

A deficiency in the decatenation checkpoint has been reported in bladder and lung cancer cells. The decatenation checkpoint was found to be impaired in all five human bladder transitional cell carcinoma lines tested (Doherty *et al*, 2003) and in three human lung cancer cell lines, whereas three other lung cancer cell lines exhibited normal decatenation checkpoint function (Nakagawa *et al*, 2004). In these studies, there was a lack of correlation between deficiency in the decatenation checkpoint and deficiency in the other cell cycle checkpoints that were assessed.

## WHY A DEFICIENCY IN STEM AND PROGENITOR CELLS?

Although it seems paradoxical that stem and progenitor cells should have a checkpoint deficiency and be susceptible to increased genome instability, certain aspects of stem cell biology must be considered in the context of cell cycle progression. Recent studies have documented distinctive properties of nuclear architecture and chromatin structure in stem cells (Azuara *et al*, 2006; Bernstein *et al*, 2006; Meshorer and Misteli, 2006). These characteristics of stemness may not be compatible with an effective decatenation checkpoint; in other words, it is possible that stem cells cannot simultaneously maintain multipotency and the decatenation checkpoint. For example, certain characteristics of stem cell chromatin might mimic the molecular stimulus of the checkpoint and would indiscriminately trigger the checkpoint if the pathway were intact. Alternatively, expression of a factor that is required for an efficient decatenation checkpoint might activate other inappropriate signalling pathways in undifferentiated cells.

Another relevant aspect of stem cell biology – independent of the first – is the heterogeneity, including the range of lifespan, of the many types of cells that are termed stem cells. The stem and progenitor cells that can be isolated and expanded in culture, such as ES cells, embryonal germ (EG) cells, neural progenitors, trophoblast stem cells, and CD34+ haematopoietic cells, are derived from cell types that normally undergo only a few cell divisions *in vivo*. In contrast, true stem cells, which have proved difficult to isolate, are long-lived populations that replenish specific cell lineages throughout the life of the organism. Transient stem and progenitor cells undergo fewer cell divisions and are at reduced risk of chromosome aberrations owing to an inefficient decatenation checkpoint and may not be under selection for an effective checkpoint; the incidence of chromosome aberrations from the deficiency will be directly proportional to the number of divisions that the cells undergo. A prediction of this model is that long-lived stem cells have an efficient decatenation checkpoint, whereas the transit amplifying stem and progenitor cells do not.

## MOLECULAR MECHANISM OF THE DECATENATION CHECKPOINT

The current understanding of the molecular control of the decatenation checkpoint is outlined in Figure 1B. Decatenation checkpoint signalling depends on ATR (ATM- and Rad3-related) kinase, as overexpression of a dominant-negative ATR prevented an efficient response (Deming *et al*, 2001). The checkpoint response does not require ATM kinase (Deming *et al*, 2001). Caffeine, which inhibits certain signalling molecules including ATR, was shown to override the G_2_ delay (Downes *et al*, 1994). ATR mediates the delay by inhibition of Plk1 (Polo-like kinase 1); Plk1 activity was reduced following ICRF-193 treatment, and conversely the checkpoint response was attenuated when active Plk1 was overexpressed (Deming *et al*, 2002). During normal cell cycle progression, Plk1 may phosphorylate cyclin B1 to cause the nuclear localisation of cyclin B1/Cdk1 complexes and promote mitosis. Expression of a constitutively nuclear cyclin B1 overrode the decatenation checkpoint (Deming *et al*, 2002).

Checkpoint function also requires BRCA1: a checkpoint defect was observed in HCC1937 cells (which have a homozygous *BRCA1* mutation) and the defect was reversed when wild-type *BRCA1* was expressed in those cells (Deming *et al*, 2001). Checkpoint activity was found to be impaired in cells from four Werner Syndrome patients and was restored when *WRN* (Werner helicase) was expressed, which indicated a role for WRN in the decatenation checkpoint (Franchitto *et al*, 2003). Werner helicase may be required for the phosphorylation of BRCA1 in response to entangled chromatids (Franchitto *et al*, 2003).

The molecular stimulus that activates the decatenation checkpoint is not understood. One possibility is that a signal emanates from sites of active decatenation, and mitotic entry is delayed until the signal abates. It has been proposed that two factors that are involved in checkpoint signalling, BRCA1 and WRN, function in the decatenation process itself (Franchitto *et al*, 2003; Lou *et al*, 2005). A role for topo II itself in checkpoint signalling has also been suggested (Gimenez-Abian *et al*, 2000). Alternatively, factors that are not involved in decatenation could be sensitive to the degree of chromosome entanglement and initiate checkpoint signalling.

Decatenation by topo II continues until the onset of anaphase, and the poleward forces on the chromatids may be required to drive decatenation to completion (Holm *et al*, 1985; Marians, 1987; Uemura *et al*, 1987; Shamu and Murray, 1992). The decatenation checkpoint therefore monitors the approach to completion, not the full completion, of chromosome disentanglement (Downes *et al*, 1994). A similar threshold basis for checkpoint activation has been documented for other cell cycle checkpoints, notably the DNA replication checkpoint in which activation requires a certain quantity of single-stranded DNA (Branzei and Foiani, 2005).

## RELATIONSHIP WITH OTHER G_2_ PHASE CHECKPOINTS

Several cell cycle checkpoints guard entry into mitosis or monitor progression through mitosis, and each checkpoint is distinguishable from the others in terms of signalling components. Observations at the cellular and molecular levels have distinguished the decatenation and DNA damage checkpoints. Bladder and lung cancer cell lines exhibited different responses to ICRF-193 treatment and irradiation (Doherty *et al*, 2003; Nakagawa *et al*, 2004). In DM87 Indian muntjac cells, in which caffeine cannot override the delay induced by irradiation or etoposide treatment (for unknown reasons), caffeine did override the delay induced by ICRF-193 treatment (Downes *et al*, 1994). Mouse ES cells delayed entry into mitosis in response to etoposide treatment but not ICRF-193 treatment (Damelin *et al*, 2005). The results with DM87 and ES cells are particularly robust because opposite responses were observed with ICRF-193 and etoposide, both of which specifically inhibit topo II activity. If ICRF-193 does cause any DNA damage, the amount of damage is insignificant by comparison to the damage caused by topo II poisons and is below the threshold that is necessary to trigger the DNA damage checkpoint.

The decatenation checkpoint does not require ATM kinase function, as the checkpoint was intact in cells from three ataxia telangiectasia (A-T) patients with mutations in *ATM* (Deming *et al*, 2001). In contrast, the DNA damage checkpoint requires ATM and was impaired in A-T cells. Phosphorylation of the Cds1 and Chk1 effector kinases was observed in lymphoblastoid lines following DNA damage, but not after ICRF-193 treatment (Deming *et al*, 2001). In addition, Cdk1 activity was reduced in response to DNA damage but not ICRF-193 treatment. These data revealed specific differences in the signalling pathways of the decatenation and DNA damage checkpoints.

It is important to consider the decatenation checkpoint in comparison to a recently characterised ICRF-193-induced metaphase arrest (Skoufias *et al*, 2004), which is probably distinct from the decatenation checkpoint. It will be important to clarify the relationship between the decatenation checkpoint (which blocks entry into mitosis) and the metaphase arrest (during mitosis), and to determine whether the metaphase arrest is a *bona fide* checkpoint that involves active signalling or whether it reflects a physical obstacle to chromatid segregation that is imposed by chromosome entanglements (Clarke *et al*, 1993).

It has been proposed that the G_2_ delay induced by chromosome entanglements is controlled by a more general antephase checkpoint that responds to aberrant chromatin topology and is mediated by p38 MAP kinase (Mikhailov *et al*, 2004). A prophase delay can be induced by inhibitors of topo II, inhibitors of histone deacetylases, and osmotic stress. However, chemical genetics screens indicated that the decatenation checkpoint and chromatin deacetylation checkpoint are distinct (Haggarty *et al*, 2003). It is possible that p38 signalling is common to several G_2_/M checkpoints that are distinguished by other signalling events. One study proposed that p38 is an ‘early sensor’ for cell damage, although only for certain types of stress or damage (Bulavin *et al*, 2001). The antephase checkpoint was proposed to be independent of ATM and the DNA damage checkpoint yet was induced by treatments that produced large quantities of DSBs (Mikhailov *et al*, 2004); if the damage and antephase checkpoints were independent, then the damage checkpoint impairment in A-T cells (with mutation in *ATM*) would be masked by the p38-mediated response, but A-T cells consistently exhibit a checkpoint defect. The various G_2_ checkpoints, including chromatin deacetylation, DNA damage, and decatenation, may or may not involve p38 MAP kinase, but they are distinguished from one another by other molecular components. The antephase checkpoint may overlap with characterised checkpoints via p38 or may represent the p38-dependent aspect of each of the characterised checkpoints. Further studies are needed to address these issues.

## THE DECATENATION CHECKPOINT AND CANCER

Chromosome aberrations – aneuploidy, heteroploidy, and rearrangements – have been documented in nearly all tumour types and actively contribute to carcinogenesis (Sen, 2000), and such aberrations could arise via an inefficient decatenation checkpoint. The products of two tumour suppressor genes, *BRCA1* and *WRN*, have roles in decatenation checkpoint function, and mutations in either gene may increase the likelihood of cancer in part because of the checkpoint deficiency. Aneuploidy is a common feature of BRCA1-associated cancers (Chappuis *et al*, 2000). Checkpoint deficiency that is acquired during carcinogenesis, for example in the lung and bladder cancer cell lines described above, may result in additional genetic changes that contribute to tumour progression (Figure 1C).

The decatenation checkpoint has particular implications for cancer in the context of stem cells and cancer stem cells (CSCs). The CSC hypothesis stipulates that only a small subset of cells in a tumour has the potential to drive tumour growth and progression (Reya *et al*, 2001). Cancer stem cells have been isolated from leukaemias and solid tumours and share many properties with normal stem cells. Cancer stem cells could theoretically arise in many ways, one of which is the transformation of a normal stem cell. The inherent decatenation checkpoint deficiency in normal stem and progenitor cells may constitute the basis for transformation and the earliest stages of carcinogenesis (Figure 1C). In addition, it seems likely that CSCs, like their normal stem cell counterparts, have a deficiency in the decatenation checkpoint, and that this deficiency could lead to additional genetic changes and increased tumour malignancy.

One potential manifestation of the relationship between the decatenation checkpoint, stem cells, and cancer is the syndrome known as constitutional trisomy mosaicism (CTM). In patients with CTM, a specific trisomy (e.g. trisomy 8) is found in a subset of cells and a range of cell types, and the trisomy is projected back to a mitotic error in a stem or progenitor cell. The CTM phenotype varies broadly and presumably depends on the nature of the particular stem or progenitor cell in which the trisomy originally appeared. Patients with CTM have a predisposition to cancer, notably haematological malignancy (Brady *et al*, 2000). One model to explain these observations is that the trisomy in the stem or progenitor cell arises from the decatenation checkpoint deficiency in those cells. The imbalance could arise from any of a range of deficiencies or spontaneous errors, but it is appealing to consider the decatenation checkpoint because of its deficiency in wild-type stem and progenitor cells.

The checkpoint deficiency in stem and progenitor cells also reflects the potential for increased genetic instability in those cells during *ex vivo* expansion, for example for stem cell therapy (Damelin and Bestor, 2006). During expansion, stem cells are forced to undergo many more rounds of cell division than they normally do *in vivo*, and the nonphysiological expansion may amplify inherent susceptibilities. Recurrent chromosome imbalances have been documented in human and mouse embryonic stem cells during laboratory culture (reviewed in Damelin and Bestor, 2006). The introduction into a patient of a stem cell that has acquired a genetic change could increase the patient's risk of cancer. Sources of genetic instability in stem cells during *ex vivo* expansion must be understood in order to minimise the risks involved (Kaufmann, 2006).

The decatenation checkpoint deficiency in lung and bladder cancer cell lines and in stem and progenitor cells suggests that factors that mediate the checkpoint could be useful targets for chemotherapy. The clinical potential was highlighted by the provocative finding that two lung cancer cell lines with a deficient decatenation checkpoint exhibited increased sensitivity to ICRF-193 (Nakagawa *et al*, 2004). Topoisomerase II poisons are commonly used to treat certain cancers, but these inhibitors target all dividing cells and are highly toxic. In contrast, bisdioxopiperazines and other compounds that target the decatenation checkpoint (Haggarty *et al*, 2003) may selectively target cancer cells in some tumours. Risk–benefit analysis will be necessary, as one potential side effect of targeting the decatenation checkpoint could be the stimulation of chromosome imbalances in otherwise normal stem and progenitor cells.

## References

[bib1] Andreassen PR, Lohez OD, Margolis RL (2003) G_2_ and spindle assembly checkpoint adaptation, and tetraploidy arrest: implications for intrinsic and chemically induced genomic instability. Mutat Res 532: 245–253 14643440 10.1016/j.mrfmmm.2003.08.020

[bib2] Azuara V, Perry P, Sauer S, Spivakov M, Jorgensen HF, John RM, Gouti M, Casanova M, Warnes G, Merkenschlager M, Fisher AG (2006) Chromatin signatures of pluripotent cell lines. Nat Cell Biol 8: 532–538 16570078 10.1038/ncb1403

[bib3] Bernstein BE, Mikkelsen TS, Xie X, Kamal M, Huebert DJ, Cuff J, Fry B, Meissner A, Wernig M, Plath K, Jaenisch R, Wagschal A, Feil R, Schreiber SL, Lander ES (2006) A bivalent chromatin structure marks key developmental genes in embryonic stem cells. Cell 125: 315–326 16630819 10.1016/j.cell.2006.02.041

[bib4] Brady AF, Waters CS, Pocha MJ, Brueton LA (2000) Chronic myelomonocytic leukaemia in a child with constitutional partial trisomy 8 mosaicism. Clin Genet 58: 142–146 11005148 10.1034/j.1399-0004.2000.580209.x

[bib5] Branzei D, Foiani M (2005) The DNA damage response during DNA replication. Curr Opin Cell Biol 17: 568–575 16226452 10.1016/j.ceb.2005.09.003

[bib6] Bulavin DV, Higashimoto Y, Popoff IJ, Gaarde WA, Basrur V, Potapova O, Appella E, Fornace Jr AJ (2001) Initiation of a G_2_/M checkpoint after ultraviolet radiation requires p38 kinase. Nature 411: 102–107 11333986 10.1038/35075107

[bib7] Cervantes RB, Stringer JR, Shao C, Tischfield JA, Stambrook PJ (2002) Embryonic stem cells and somatic cells differ in mutation frequency and type. Proc Natl Acad Sci USA 99: 3586–3590 11891338 10.1073/pnas.062527199 PMC122567

[bib8] Chappuis PO, Nethercot V, Foulkes WD (2000) Clinico-pathological characteristics of BRCA1- and BRCA2-related breast cancer. Semin Surg Oncol 18: 287–295 10805950 10.1002/(sici)1098-2388(200006)18:4<287::aid-ssu3>3.0.co;2-5

[bib9] Clarke DJ, Johnson RT, Downes CS (1993) Topoisomerase II inhibition prevents anaphase chromatid segregation in mammalian cells independently of the generation of DNA strand breaks. J Cell Sci 105(Part 2): 563–569 8408285 10.1242/jcs.105.2.563

[bib10] Damelin M, Bestor TH (2006) Decatenation checkpoint deficiency destabilizes the stem cell genome. Cell Cycle 5: 345–346 16479155 10.4161/cc.5.4.2480

[bib11] Damelin M, Sun YE, Brundula Sodja V, Bestor TH (2005) Decatenation checkpoint deficiency in stem and progenitor cells. Cancer Cell 8: 479–484 16338661 10.1016/j.ccr.2005.11.004

[bib12] Deming PB, Cistulli CA, Zhao H, Graves PR, Piwnica-Worms H, Paules RS, Downes CS, Kaufmann WK (2001) The human decatenation checkpoint. Proc Natl Acad Sci USA 98: 12044–12049 11593014 10.1073/pnas.221430898 PMC59764

[bib13] Deming PB, Flores KG, Downes CS, Paules RS, Kaufmann WK (2002) ATR enforces the topoisomerase II-dependent G_2_ checkpoint through inhibition of Plk1 kinase. J Biol Chem 277: 36832–36838 12147700 10.1074/jbc.M206109200

[bib14] Doherty SC, McKeown SR, McKelvey-Martin V, Downes CS, Atala A, Yoo JJ, Simpson DA, Kaufmann WK (2003) Cell cycle checkpoint function in bladder cancer. J Natl Cancer Inst 95: 1859–1868 14679155 10.1093/jnci/djg120

[bib15] Downes CS, Clarke DJ, Mullinger AM, Gimenez-Abian JF, Creighton AM, Johnson RT (1994) A topoisomerase II-dependent G_2_ cycle checkpoint in mammalian cells. Nature 372: 467–470 7984241 10.1038/372467a0

[bib16] Franchitto A, Oshima J, Pichierri P (2003) The G_2_-phase decatenation checkpoint is defective in Werner syndrome cells. Cancer Res 63: 3289–3295 12810661

[bib17] Gimenez-Abian JF, Clarke DJ, Devlin J, Gimenez-Abian MI, De la Torre C, Johnson RT, Mullinger AM, Downes CS (2000) Premitotic chromosome individualization in mammalian cells depends on topoisomerase II activity. Chromosoma 109: 235–244 10968252 10.1007/s004120000065

[bib18] Gorbsky GJ (1994) Cell cycle progression and chromosome segregation in mammalian cells cultured in the presence of the topoisomerase II inhibitors ICRF-187 [(+)-1,2-bis(3,5-dioxopiperazinyl-1-yl)propane; ADR-529] and ICRF-159 (Razoxane). Cancer Res 54: 1042–1048 8313360

[bib19] Haggarty SJ, Koeller KM, Kau TR, Silver PA, Roberge M, Schreiber SL (2003) Small molecule modulation of the human chromatid decatenation checkpoint. Chem Biol 10: 1267–1279 14700634 10.1016/j.chembiol.2003.11.014

[bib20] Holm C, Goto T, Wang JC, Botstein D (1985) DNA topoisomerase II is required at the time of mitosis in yeast. Cell 41: 553–563 2985283 10.1016/s0092-8674(85)80028-3

[bib21] Ishida R, Sato M, Narita T, Utsumi KR, Nishimoto T, Morita T, Nagata H, Andoh T (1994) Inhibition of DNA topoisomerase II by ICRF-193 induces polyploidization by uncoupling chromosome dynamics from other cell cycle events. J Cell Biol 126: 1341–1351 8089169 10.1083/jcb.126.6.1341 PMC2290951

[bib22] Kaufmann WK, Kies PE (1998) DNA signals for G2 checkpoint response in diploid human fibroblasts. Mutat Res 400: 153–167 9685622 10.1016/s0027-5107(98)00041-4

[bib23] Kaufmann WK (2006) Dangerous entanglements. Trends Mol Med 12: 235–237 16631407 10.1016/j.molmed.2006.04.001

[bib24] Lou Z, Minter-Dykhouse K, Chen J (2005) BRCA1 participates in DNA decatenation. Nat Struct Mol Biol 12: 589–593 15965487 10.1038/nsmb953

[bib25] Marians KJ (1987) DNA gyrase-catalyzed decatenation of multiply linked DNA dimers. J Biol Chem 262: 10362–10368 3038875

[bib26] Meshorer E, Misteli T (2006) Chromatin in pluripotent embryonic stem cells and differentiation. Nat Rev Mol Cell Biol 7: 540–546 16723974 10.1038/nrm1938

[bib27] Mikhailov A, Shinohara M, Rieder CL (2004) Topoisomerase II and histone deacetylase inhibitors delay the G_2_/M transition by triggering the p38 MAPK checkpoint pathway. J Cell Biol 166: 517–526 15302851 10.1083/jcb.200405167 PMC2172207

[bib28] Nakagawa T, Hayashita Y, Maeno K, Masuda A, Sugito N, Osada H, Yanagisawa K, Ebi H, Shimokata K, Takahashi T (2004) Identification of decatenation G_2_ checkpoint impairment independently of DNA damage G_2_ checkpoint in human lung cancer cell lines. Cancer Res 64: 4826–4832 15256452 10.1158/0008-5472.CAN-04-0871

[bib29] Reya T, Morrison SJ, Clarke MF, Weissman IL (2001) Stem cells, cancer, and cancer stem cells. Nature 414: 105–111 11689955 10.1038/35102167

[bib30] Roca J, Ishida R, Berger JM, Andoh T, Wang JC (1994) Antitumor bisdioxopiperazines inhibit yeast DNA topoisomerase II by trapping the enzyme in the form of a closed protein clamp. Proc Natl Acad Sci USA 91: 1781–1785 8127881 10.1073/pnas.91.5.1781 PMC43247

[bib31] Sen S (2000) Aneuploidy and cancer. Curr Opin Oncol 12: 82–88 10687734 10.1097/00001622-200001000-00014

[bib32] Shamu CE, Murray AW (1992) Sister chromatid separation in frog egg extracts requires DNA topoisomerase II activity during anaphase. J Cell Biol 117: 921–934 1315785 10.1083/jcb.117.5.921 PMC2289485

[bib33] Skoufias DA, Lacroix FB, Andreassen PR, Wilson L, Margolis RL (2004) Inhibition of DNA decatenation, but not DNA damage, arrests cells at metaphase. Mol Cell 15: 977–990 15383286 10.1016/j.molcel.2004.08.018

[bib34] Uemura T, Ohkura H, Adachi Y, Morino K, Shiozaki K, Yanagida M (1987) DNA topoisomerase II is required for condensation and separation of mitotic chromosomes in *S. pombe*. Cell 50: 917–925 3040264 10.1016/0092-8674(87)90518-6

[bib35] Vagnarelli P, Morrison C, Dodson H, Sonoda E, Takeda S, Earnshaw WC (2004) Analysis of Scc1-deficient cells defines a key metaphase role of vertebrate cohesin in linking sister kinetochores. EMBO Rep 5: 167–171 14749720 10.1038/sj.embor.7400077 PMC1298988

